# Prevalence of heart failure in Australia: a systematic review

**DOI:** 10.1186/s12872-016-0208-4

**Published:** 2016-02-06

**Authors:** Berhe W. Sahle, Alice J. Owen, Mutsa P. Mutowo, Henry Krum, Christopher M. Reid

**Affiliations:** Centre of Cardiovascular Research and Education in Therapeutics, Department of Epidemiology and Preventive Medicine, Monash University, 99 Commercial Road, Melbourne, Vic 3004 Australia; School of Public Health, Mekelle University, Mekelle, Ethiopia; School of Public Health, Department of Epidemiology and Preventive Medicine, Monash University, Melbourne, Australia; School of Public Health, Curtin University, Perth, Australia

**Keywords:** Heart failure, Incidence, Prevalence, Indigenous, Australia

## Abstract

**Background:**

In the absence of a systematic collection of data pertaining to heart failure, summarizing the data available from individual studies provides an opportunity to estimate the burden of heart failure. The present study systematically reviewed the literature to estimate the incidence and prevalence rates of heart failure in Australia.

**Methods:**

Studies reporting on prevalence or incidence of heart failure published between 1990 and 2015 were identified through a systematic search of Embase, PubMed, Ovid Medline, MeSH, Scopus and websites of the Australian Institute of Health, and Welfare and Australian Bureau of Statistics.

**Results:**

The search yielded a total of 4978 records, of which thirteen met the inclusion criteria. There were no studies reporting on the incidence of heart failure. The prevalence of heart failure in the Australian population ranged between 1.0 % and 2.0 %, with a significant proportion of cases being previously undiagnosed. The burden of heart failure was higher among Indigenous than non-Indigenous Australians (age-standardized prevalence rate ratio of 1.7). Heart failure was prevalent in women than men, and in rural and remote regions than in the metropolitan and capital territories.

**Conclusion:**

This systematic review highlights the limited available data on the epidemiology of heart failure in Australia. Population level studies, using standardized approaches, are needed in order to precisely describe the burden of HF in the population.

**Electronic supplementary material:**

The online version of this article (doi:10.1186/s12872-016-0208-4) contains supplementary material, which is available to authorized users.

## Background

Heart failure (HF) causes a significant burden for patients and healthcare systems in developed countries [1, 2]. Approximately 50–75 % of patients with HF die within five years of diagnosis [3, 4]. Evidence shows that HF accounts for 1–3 % of overall healthcare spending, mainly due to repeated hospital admissions and prolonged inpatient length of stay [1]. In developed countries, prevalence of HF ranges from 1–3 %, rising to 10 % or more in those aged 75 years or older [1, 2], while estimates in developing countries are not routinely reported.

The relationship between obesity, hypertension, smoking and cholesterol with cardiovascular morbidity is undisputed. Australia has made significant attempts to address risk factors contributing to the onset of chronic diseases [5]. Public health policy on tobacco control, improvements in surgical treatment and the widespread use of lipid and blood pressure-lowering drugs have resulted in a significant decline in the burden of major cardiovascular diseases [5–8]. Despite these improvements, the burden of diabetes and obesity in the population has steadily increased over time [7].

Data on the incidence of HF is not reported in the Australian Institute of Health and Welfare (AIHW) periodic statistics [9], while the Australian National Health Surveys (NHS) report prevalence rates based on self-report [10]. However, self-reported data may underestimate the true burden of HF because the early stages of HF may be only mildly symptomatic; therefore many people may be unaware that they have the condition [7]. Similarly, estimation based on mortality data may under-report the actual burden of HF, as the international classification of diseases (ICD) recommends coding underlying causes of death such as ischemic heart diseases rather than HF, which is often the terminal illness of other cardiovascular diseases [10, 11]. Variation in the definition of HF and process of estimation therefore limit the validity and utility of data from these sources [9, 10].

Despite the morbidity and economic burden to the community and poor patient outcomes, there is a deficiency in the systematic collection of data pertaining the incidence and prevalence of HF. Therefore, summarizing the data available from individual studies provides an opportunity to estimate the burden of HF. The present study systematically reviewed the literature to estimate the incidence and prevalence rates of heart failure in Australia.

## Methods

### Literature search

The process of the review was designed and undertaken according to the Preferred Reporting Items for Systematic Reviews and Meta-Analyses (Additional file 1) guidelines [12]. We searched Embase, PubMed, Ovid Medline, MeSH, and Scopus for articles published under subject terms: heart failure, cardiac failure, congestive heart failure, chronic heart failure, left ventricular dysfunction, systolic heart failure, diastolic heart failure combined with epidemiology, incidence, prevalence, burden and morbidity. The search was limited to articles published in the Australian population in English, between September 1990 and 20 July 2015 (Additional file 2: Table S1). Relevant studies on the prevalence and incidence of HF in Australia were included. We further searched the AIHW and Australian Bureau of Statistics (ABS) websites to identify relevant datasets and reports.

### Study selection

**Fig. 1:**
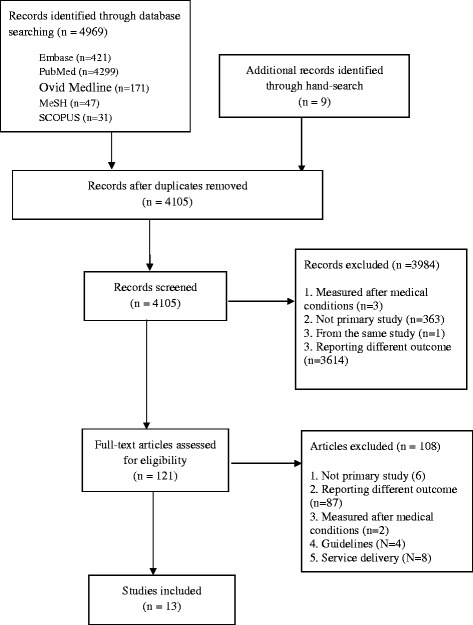
PRISMA flow diagram of selection process

Figure 1 summarises the selection process for the studies. Publications retrieved from all databases were organized in Endnote and duplicate publications were removed (by BW). Two reviewers (BWS and MPM) independently reviewed abstracts and full text citations to screen for eligibility. The reviewers had no difference regarding to the selection of eligible publications. Studies were eligible if: (1) reported on prevalence or incidence of HF in Australia; (2) not clinical trials or therapeutic studies; (3) and conducted on patients with pre-existing medical conditions such as hypertension and coronary heart disease.

### Data extraction and summarisation

A summary table was prepared to ensure that all relevant data including type of study, sample size, sampling methods, age and sex of the participants, year of study, scope of the study (national or single center), method of diagnosis, and the primary outcomes (incidence and prevalence rates) were extracted. Tables 1 and 2 provides a summary of the studies included in the review. Due to heterogeneity in data sources and design of the individual studies, data were summarised narratively.

**Table 1:** Studies reporting prevalence of heart failure

				Prevalence (%)			
Author	Participants	Type of study	Method of diagnosis	Male	Female	Total	Quality rating
Abhayaratna 2006 [19]	1275 random sample, aged 60–86 years	Cross sectional	physical examination, and Echo	8.2	4.4	6.3	4
Knox 2008 [17]	9156 of all ages, selected from 305 GP centers	Multi-center Cross sectional	Medical record	-	-	1.8	4.1
Harrison 2013 [18]	8707 of all ages selected from 290 GP centers	Multi-center, Cross sectional	Medical record	-	-	1.5	4
NHS 2004/5 [14]	National survey on ≥18 y	National survey	Self-report	0.9	1.8	1.4	3
NHS 2007/8 [15]	National survey on ≥18 y	National survey	Self-report	1.0	1.7	1.3	3
NHS 2011/12 [16]	National survey on ≥18 y	National survey	Self-report	1.0	1.5	1.2	3
NATSIHS 2008 [22]	National survey on ≥18 y aboriginals	Survey	Self-report	1.74	2.88	1.0	
McGrady 2012 [21]	436 random sample >18 yrs	Cross sectional	Echo	7	4	5.3	4.3
Clark 2004 [26]	No mentioned	Cross sectional	administrative data	-	-	2.0	3.0
Clark 2005 [23]	No mentioned	Cross sectional	administrative data	-	-	1.8	3.3
Clark 2007 [20]	23845, ≥60 yrs selected from 341 general practice	Cross sectional	WHO criteria	-	-	13.1	3.5

*CHF* congestive heart failure, *GP* general practice, *NHS* national health survey, *WHO* World Health Organization, *NATSIHS* national aboriginal and torres strait islander health survey, *Echo* echocardiography

**Table 2:** Studies reporting prevalence of heart failure by geographic locations

Author	Sample size	Geographic stratum (%)	Quality rating
Clark 2004 [26]	Not mentioned	Central and west Australia (28.8–79.9 per 1000)	3.2
		Queensland, New South Wales, Western Australia (20.3–28.7 per 1000)	
Clark 2005 [23]	Not mentioned	Australian Capital Territory (14 per 1000)	3.3
		Northern Territory (29.5 per 1000)	
McGrady 2012	436 random sample >18 yrs	Urban (6 %) Remote (4 %)	4.3
Clark 2007 [20]	23845, ≥60 yrs selected from 341 GP center	Capital cities and metropolitan (12.4 %)	3.5
		Large and small rural towns (16.1 %)	
		Other rural and remote areas (13.9 %)	

### Quality assessment

Quality of the studies selected for the review were assessed using a checklist comprising the items recommended by Sanderson et al. [13]. The items in the checklist include: appropriate source population; measurement methods; methods outlined to deal with any design-specific issues such as recall bias, interviewer bias, biased loss to follow or blinding; design and/or analytical methods; and appropriate use of statistics for primary analysis of effect. Each criterion was given a score of zero or one, which was then summed in to summary score (0 to 5). The score for each article was an average value of the score assigned by two independent reviewers (BWS and DM Emdadul Hoque). The studies selected for the review had an overall quality score of three or more out of five. This study used publicly available data which does not require ethical approval.

## Results

### Description of studies

The search yielded 4978 citations. After screening the titles and abstracts and removing duplicates, 121 studies remained for the full text review. Thirteen studies which met the inclusion criteria were selected (Tables 1 and 2).Ten were cross-sectional studies [14–23], and three studies extrapolated data from other countries to the Australian population [24–26].

### Participants and method of diagnosis

Six studies recruited participants from the community [14–16, 19, 21, 22] three from clinical settings [17, 18, 20] and the remainder used administrative data [14, 23, 25, 26]. Five studies were conducted in adults (≥18 years) [14–16, 22], two in those 60 years or over [19, 20], two in all age groups [17, 18], with participant’s age not specified in the reminder [23–26]. Heart failure was defined based self-report [14–16, 22], echocardiography (and physical assessment) [19, 21], using modified World Health Organization criteria [20] while the rest used administrative data [17, 18, 23, 25, 26] (Tables 1 and 2).

### Incidence of heart failure

There were no studies reporting the incidence of HF in Australia. According to the AHIW (based up on extrapolation of incidence rates from large scale international cohorts to the Australian population characteristics) an estimated 30,000 new cases of HF are diagnosed each year in Australia [24], which translates in to a crude incidence rate of 2.1 per 1000 population.

### Prevalence of heart failure

Prevalence of HF was reported in twelve studies [14–23, 25, 26]. Based on self-report, the prevalence of HF and oedema in adults was estimated to be 1.4 % in the 2004/5, 1.3 % in the 2007/8, and 1.2 % in the 2011/12 National Health Surveyes [14–16].

The national population prevalence of HF was reported in four studies, ranging from 1.5–2.0 % [17, 18, 20, 26]. Two studies reported the prevalence of chronic conditions among a sample of 9156 [17] and 8707 [18] general practice patients adjusted for the Australian population. In these studies the prevalence of HF in the population was estimated to be 1.8 % [17] and 1.5 % [18], respectively. In the third study, HF data were derived by applying international HF prevalence data to the Australian population [25]. The study reported a national prevalence of 17.9 per 1000 [25]. Slightly higher prevalence rate (2.0 %) was also reported in a similar study, which extrapolated HF data from Scotland and United Kingdom to the Australian popaultion [26].

In the Canberra Heart Study, which screened 1275 people aged 60–86 years old, 6.7 % (95 % CI: 4.4 %-7.1 %) of them had HF and 0.6 % (95 % CI: 0.3 %-1.2 %) of these were previously undiagnosed [19]. In the same study, the prevalence of HF ranged between 3.1 % in those 60–64 years old to 13.6 % in those 80–86 years old [19]. In another study on a sample of 23845 general practice patients aged 60 years and over, HF was reported in 13.1 % of them comprising previously diagnosed cases (prevalence, 11.2 %) and newly diagnosed cases (prevalence, 1.9 %) [20] However, this rate was not adjusted for the general population.

In a study that screened 436 indigenous adults (18 years or over), HF was diagnosed in 5.3 % of them, of which 65 % were previously undiagnosed [21]. In the 2004/5 National Aboriginal and Torres Strait Islander Health Survey (NATSIHS) [22] the prevalence of HF among Indigenous Australians was 1.0 %. However, following age-standardization the prevalence of HF was 1.7 times higher among Indigenous than non-Indigenous Australians [22].

### Heart failure by gender

In the NHS [14–16, 22] HF was almost twice common in women than in men. Prevalence rates were 0.89 % vs 1.8 % in the 2004/5 NHS, 1.0 % vs 1.7 % in the NHS 2007/8 and 1.0 % vs 1.5 % in the NHS 2011/12 in men and women, respectively [14–16]. Similarly, in the 2004/5 NATSIHS, the age-standardized prevalence of HF was 17.4 per 1000 in men and 28.8 per 1000 in women [22]. On the other hand, In the Canberra Heart Study, HF was twice as common in men (8.2 %) than in women (4.4 %) [19]. Similarly, the prevalence of HF was 7.0 % in male and 4.0 % in female adult Indigenous Australians [21].

### Heart failure by geographic location

Four studies reported on HF by geographic location [20, 21, 23, 25]. Clark and colleagues reported a higher prevalence of HF in large and small rural towns (16.1 %) than in the capital city and metropolitan areas (12.4 %) [20]. In the second study, prevalence rates ranged between 2.9 % and 7.8 % in Central and Western Australia and between 2.0 % and 2.9 % in parts of Queensland, New South Wales, and Western Australia [23]. The prevalence of HF also varied from 14 per 1000 in the Australian Capital Territory compared to 29 per 1000 in rural Northern Territory [25]. McGrady and colleagues also reported higher in prevalence of HF in urban Aboriginals (6.0 %) than those in remote communities (4.0 %) [21].

## Discussion

Our findings indicate that the prevalence of HF in the Australian population ranges from 1.0–2.0 %, and varies by Indigenous status, gender, age, and geographic location. The burden of HF was higher among Indigenous than non-Indigenous Australians, and in rural and remote than in capital city and metropolitan areas. In most of the studies reviewed, HF was more prevalent in women than in men.

As indicated in the AIHW reports, there are no studies reporting on the incidence of HF. [7] The only data available was the number of new cases of HF diagnosed each year, estimated based on overseas data, which may not be generalizable to the population due to limitations inherent to the process of extrapolation [24].

Our findings of the prevalence of HF in Australia (1.0–2.0 %) is congruent with data from Europe and North America where prevalence of HF range between 1.3 % and 2.2 % [1, 27–29]. Consistent with existing knowledge [30, 31] HF was three or more times higher in the elderly than in the population. In general, the NHS [14–16] reported a lower prevalence of HF compared with the individual studies [17, 18, 20, 26] which may be related to the use of self-reported diagnosis in the NHS [7]. The significant proportion of previously undiagnosed HF patients [19, 21] also reveal a chance of underestimation when HF is defined based on self-reported diagnosis.

In most of the studies [14–16, 22] HF was twice more prevalent in women than men, although men were more affected than women in two other studies [19, 21]. The inconsistency in the prevalence of HF by gender has been reported in several studies [3, 32, 33]. In Australia, major risk factors of HF including coronary heart diseases, diabetes, and high blood pressure are higher in men than in women [7, 34]. However, risk factors of HF such as diabetes, hypertension, and left ventricular dysfunction have stronger risk of progression to HF in women than men, which may explain the higher prevalence of HF among women [30, 35]. The higher burden of HF among women could be also be attributed to the longer life expectancy and higher incidence of HF in later years of life in women than men [36].

The higher prevalence of HF among Indigenous Australians is consistent with the existing literature. The latest Australian statistics shows that cardiovascular diseases are 1.3 times more common among Indigenous than their non-Indigenous counterparts [8]. The excess burden of HF, cardiovascular diseases in general, in Indigenous population is related to the overall disparity in the social and economic status that impact on their health status [22]. The risk factors of HF are also reported to be more prevalent among Indigenous population than the nationally reported data [21].

According to previous studies [22, 25] the disproportionately higher burden of HF in rural and remote towns is related to the concentration of older age inhabitants and higher prevalence of socio-demographic risk factors in these regions. Indigenous Australians, who have higher prevalence of HF also concentrate in rural and remote towns [25]. The comparatively lower access to healthcare services in rural and remote regions, which may affect early identification and management of the risk factors predisposing to HF, could also contribute to the geographic disparity [20, 23, 25].

The syndrome of HF has different causes and diverse clinical presentations, and long-term clinical evolution that makes its diagnosis challenging [1, 37]. Studies based on self-reported diagnosis are likely to underestimate the burden of HF because participants may not know their HF status [37, 38]. Limitations inherent to use of administrative data such misclassification may also compromise the validity of HF data from such studies. Therefore the overall estimate of the burden of HF is likely to be affected by the use of different definitions and measurement methods for HF.

This systematic review has some limitations. There were limited studies on epidemiology of HF in Australia and therefore studies that used varying HF definition (such as heart failure and oedema) were included in the review. Due to the limited number of studies, further description of the pattern of HF over time was not possible. Moreover, the search was restricted to PubMed indexed journals, and unpublished reports. As a result, articles published non-PubMed indexed journals may have been missed. Despite these limitations, the current review still provides valuable information on the prevalence of HF in the Australian population. Extensive and systematic literature search using pre-defined quality assessment and inclusion criteria were employed.

## Conclusions

This review shows that the prevalence heart failure in Australia is similar to other developed countries, however it has a significant burden in specific demographic profiles in the country, namely Indigenous groups, females and those who reside in rural and remote areas. Further population-level studies, with clearly defined method of diagnosis, are needed for a more accurate description of the burden of HF in Australia.

## Additional files

Additional file 1:
**PRISMA guideline for reporting systematic reviews.** (DOCX 17 kb) Additional file 2:
**Literature search history: a sample using Ovid MEDLINE.** (DOC 63 kb)
