# Supplementary material for: Prevalence of heart failure in Australia: a systematic review
Source: BMC Cardiovasc Disord. 2016 Feb 6;16:32. doi: 10.1186/s12872-016-0208-4 (PMC4744379; doi:10.1186/s12872-016-0208-4)
Supplement: Additional file 1: — PRISMA guideline for reporting systematic reviews. (DOCX 17 kb) [file 12872_2016_208_MOESM1_ESM.docx]

***Attachment 1***: Literature search history

Database(s): Ovid MEDLINE(R) 1946 to Present with Daily Update
Search Strategy:

| **#** | **Searches** | **Results** |
| --- | --- | --- |
| 1 | heart failure.mp. [mp=title, abstract, original title, name of substance word, subject heading word, keyword heading word, protocol supplementary concept word, rare disease supplementary concept word, unique identifier] | 140554 |
| 2 | limit 1 to yr="1990 - 2015" | 111349 |
| 3 | cardiac failure.mp. [mp=title, abstract, original title, name of substance word, subject heading word, keyword heading word, protocol supplementary concept word, rare disease supplementary concept word, unique identifier] | 9968 |
| 4 | limit 3 to yr="1990 - 2015" | 7000 |
| 5 | congestive heart failure.mp. [mp=title, abstract, original title, name of substance word, subject heading word, keyword heading word, protocol supplementary concept word, rare disease supplementary concept word, unique identifier] | 32811 |
| 6 | limit 5 to yr="1990 - 2015" | 24573 |
| 7 | chronic heart failure.mp. [mp=title, abstract, original title, name of substance word, subject heading word, keyword heading word, protocol supplementary concept word, rare disease supplementary concept word, unique identifier] | 11352 |
| 8 | limit 7 to yr="1990 - 2015" | 10706 |
| 9 | left ventricular dysfunction.mp. [mp=title, abstract, original title, name of substance word, subject heading word, keyword heading word, protocol supplementary concept word, rare disease supplementary concept word, unique identifier] | 9226 |
| 10 | limit 9 to yr="1990 - 2015" | 8074 |
| 11 | systolic heart failure.mp. [mp=title, abstract, original title, name of substance word, subject heading word, keyword heading word, protocol supplementary concept word, rare disease supplementary concept word, unique identifier] | 1423 |
| 12 | limit 11 to yr="1990 - 2015" | 1423 |
| 13 | diastolic heart failure.mp. [mp=title, abstract, original title, name of substance word, subject heading word, keyword heading word, protocol supplementary concept word, rare disease supplementary concept word, unique identifier] | 977 |
| 14 | limit 13 to yr="1990 - 2015" | 974 |
| 15 | incidence.mp. [mp=title, abstract, original title, name of substance word, subject heading word, keyword heading word, protocol supplementary concept word, rare disease supplementary concept word, unique identifier] | 598901 |
| 16 | limit 15 to yr="1990 - 2015" | 501034 |
| 17 | prevalence.mp. [mp=title, abstract, original title, name of substance word, subject heading word, keyword heading word, protocol supplementary concept word, rare disease supplementary concept word, unique identifier] | 454618 |
| 18 | limit 17 to yr="1990 - 2015" | 422300 |
| 19 | burden.mp. [mp=title, abstract, original title, name of substance word, subject heading word, keyword heading word, protocol supplementary concept word, rare disease supplementary concept word, unique identifier] | 109680 |
| 20 | limit 19 to yr="1990 - 2015" | 104349 |
| 21 | morbidity.mp. [mp=title, abstract, original title, name of substance word, subject heading word, keyword heading word, protocol supplementary concept word, rare disease supplementary concept word, unique identifier] | 255160 |
| 22 | limit 21 to yr="1990 - 2014" | 222933 |
| 23 | 2 or 4 or 6 or 8 or 10 or 12 or 14 | 120439 |
| 24 | 16 or 18 or 20 or 22 | 1118975 |
| 25 | Australia.mp. [mp=title, abstract, original title, name of substance word, subject heading word, keyword heading word, protocol supplementary concept word, rare disease supplementary concept word, unique identifier] | 109890 |
| 26 | 23 and 24 | 21597 |
| 27 | 25 and 26 | 171 |
